# On the Exhalation of Bicarbonate of Ammonia by the Lungs

**Published:** 1847-06

**Authors:** Lewis Thompson


*On the Exhalation of Bicarbonate of Ammonia by the Lungs.* By Lewis Thompson, M. R. C. S. &c.—H.aving lately had occasion to ascertain the amount of moisture given off by the lungs of several healthy individuals during a fixed period, I was induced to examine the nature of the fluid thus condensed.

The result has proved that bicarbonate of ammonia, is constantly exhaled from the lungs, to the extent of rather more than three grains every twenty-four hours for each individual; and although this quantity may appear trifling, yet the amount arising from a large population like that of London is well worthy of notice, and must exceed 150 tons of solid bicarbonate of ammonia per annum; and if, as is extremely probable, other animals also exhale this substance, the atmosphere must not only always contain enough of this agent for the purposes of vegetation, but by a reciprocal action, the mutual increase of vegetables and animals would only tend to render the air better adapted for the due development of both. The existence of ammonia in the breath, may be easily demonstrated by respiring air that has been passed through diluted sulphuric acid, and then expiring it through a tube surrounded by water at 32° F., to the further end of which a vessel is attached to receive the fluid which condenses. On acidulating this fluid with or two drops of pure muriatic acid and evaporating to dryness on a water bath, a residue will be obtained, which, when dissolved in fire or six drops of water, and introduced into a test tube, will give off ammonia on the addition of a strong solution of potash, as evidences by its action on turmeric paper and muriatic acid, or by its peculiar smell. The respiratory process should be continued for an hour or two.

It would be interesting to know whether any difference is observable in the amount of ammonia exhaled by the lungs of individuals, suffering from disease of the kidneys, diabetes, &c.—*Philosophical Magazine and Journal.*

